# Patients with ANCA-associated vasculitis admitted to the intensive care unit with acute vasculitis manifestations: a retrospective and comparative multicentric study

**DOI:** 10.1186/s13613-017-0262-9

**Published:** 2017-04-05

**Authors:** Julien Demiselle, Johann Auchabie, François Beloncle, Philippe Gatault, Steven Grangé, Damien Du Cheyron, Jean Dellamonica, Sonia Boyer, Dimitri Titeca Beauport, Lise Piquilloud, Julien Letheulle, Christophe Guitton, Nicolas Chudeau, Guillaume Geri, François Fourrier, René Robert, Emmanuel Guérot, Julie Boisramé-Helms, Pierre Galichon, Pierre-François Dequin, Alexandre Lautrette, Pierre-Edouard Bollaert, Ferhat Meziani, Loïc Guillevin, Nicolas Lerolle, Jean-François Augusto

**Affiliations:** 1 Département de Réanimation Médicale et de Médecine Hyperbare, Centre Hospitalier Universitaire, 4 rue Larrey, 49933 Angers Cedex 9, France; 2 Néphrologie-Dialyse-Transplantation, CHU Angers, 4 rue Larrey, 49933 Angers Cedex 9, France; 3 Service de Néphrologie et Immunologie Clinique, CHRU Tours, Tours, France; 4 Medical Intensive Care Unit, Rouen University Hospital, Rouen, France; 5 Service de Réanimation Médicale, CHU de Caen, Avenue de la Côte de Nacre, CS 30001, 14033 Caen Cedex 9, France; 6 Medical Intensive Care Unit, Archet 1 University Hospital, Route de St Antoine, CS 23079, 06202 Nice, France; 7 Medical Intensive Care Unit, Amiens University Medical Center, 80054 Amiens, Cedex 1, France; 8 Service de Médecine Intensive Adulte et Centre des Brûlés, Centre Hospitalier Universitaire Vaudois, Lausanne, Switzerland; 9 Service de Réanimation Médicale, Hôpital Pontchaillou, CHU Rennes, 2 rue Henri Le Guilloux, 35033 Rennes Cedex, France; 10 Medical Intensive Care Unit, Hôtel-Dieu, University Hospital of Nantes, 30 bd Jean Monnet, 44093 Nantes, France; 11 UMR 1064, Inserm, 30 bd Jean Monnet, 44093 Nantes, France; 12 Service de Reanimation Medico-Chirurgicale, Centre Hospitalier du Mans, 194 Avenue Rubillard, 72037 Le Mans, France; 13 Service de Réanimation Médicale, Hôpital Cochin, Paris, France; 14 Réanimation, Centre de Réanimation Polyvalente, Hôpital Roger Salengro, CHRU de Lille, Lille, France; 15 Service de Réanimation Médicale, CHU de Poitiers, Poitiers, France; 16 Service de Réanimation Médicale, Hôpital Européen Georges Pompidou, Paris, France; 17 Service de Réanimation Médicale, Nouvel Hôpital Civil, Hôpitaux Universitaires de Strasbourg, Strasbourg, France; 18 EA 7293, Fédération de Médecine Translationnelle de Strasbourg (FMTS), Faculté de Médecine, Université de Strasbourg, Strasbourg, France; 19 APHP, Hôpital Tenon, Urgences Néphrologiques et Transplantation Rénale, Paris, France; 20 Service de Réanimation Polyvalente, Hôpital Bretonneau, Tours, France; 21 Service de Réanimation Médicale Polyvalente, CHU Gabriel Montpied, 58 rue Montalembert, 63000 Clermont-Ferrand, France; 22 Service de Réanimation Médicale, CHU de Nancy Hôpital Central, 29 Avenue de Lattre de Tassigny, 54035 Nancy Cedex, France; 23 Département de Médecine Interne, Assistance Public des Hôpitaux de Paris, Hôpital Cochin, Paris, France

**Keywords:** Anti-neutrophil cytoplasmic antibody, ANCA-associated vasculitis, Intensive care unit, Mortality

## Abstract

**Purpose:**

Data for ANCA-associated vasculitis (AAV) patients requiring intensive care are scarce.

**Methods:**

We included 97 consecutive patients with acute AAV manifestations (new onset or relapsing disease), admitted to 18 intensive care units (ICUs) over a 10-year period (2002–2012). A group of 95 consecutive AAV patients with new onset or relapsing disease, admitted to two nephrology departments with acute vasculitis manifestations, constituted the control group.

**Results:**

In the ICU group, patients predominantly showed granulomatosis with polyangiitis and proteinase-3 ANCAs. Compared with the non-ICU group, the ICU group showed comparable Birmingham vasculitis activity score and a higher frequency of heart, central nervous system and lungs involvements. Respiratory assistance, renal replacement therapy and vasopressors were required in 68.0, 56.7 and 26.8% of ICU patients, respectively. All but one patient (99%) received glucocorticoids, 85.6% received cyclophosphamide, and 49.5% had plasma exchanges as remission induction regimens. Fifteen (15.5%) patients died during the ICU stay. The following were significantly associated with ICU mortality in the univariate analysis: the need for respiratory assistance, the use of vasopressors, the occurrence of at least one infection event in ICU, cyclophosphamide treatment, sequential organ failure assessment at admission and simplified acute physiology score II. After adjustment on sequential organ failure assessment or infection, cyclophosphamide was no longer a risk factor for mortality. Despite a higher initial mortality rate of ICU patients within the first hospital stay (*p* < 0.0001), the long-term mortality of hospital survivors did not differ between ICU and non-ICU groups (18.6 and 20.4%, respectively, *p* = 0.36). Moreover, we observed no renal survival difference between groups after a 1-year follow-up (82.1 and 80.5%, *p* = 0.94).

**Conclusion:**

This study supports the idea that experiencing an ICU challenge does not impact the long-term prognosis of AAV patients.

**Electronic supplementary material:**

The online version of this article (doi:10.1186/s13613-017-0262-9) contains supplementary material, which is available to authorized users.

## Background

Anti-neutrophil cytoplasmic antibodies (ANCAs)-associated vasculitis (AAV) are life-threatening multisystem autoimmune diseases characterized by necrotizing inflammation of small- to medium-sized vessels [1, 2]. There are three differentiated entities based on clinical and pathological criteria: microscopic polyangiitis (MPA), granulomatosis with polyangiitis (GPA) and eosinophilic granulomatosis with polyangiitis (EGPA) [3]. Their clinical spectrum partially overlaps. Indeed, rapidly progressive glomerulonephritis is the typical renal presentation of MPA and GPA, but is rarely present in EGPA [1]. Diffuse alveolar hemorrhage (DAH) is the most critical lung injury observed with all entities, but more frequently with MPA and GPA [4, 5]. Other respiratory presentations include pulmonary infiltrates and nodules, the latter being observed predominantly in GPA [6]. Even though ANCA negativity does not exclude AAV diagnosis, diffuse forms of AAV are usually associated with serum positivity for ANCAs [1, 7]. Given their high level of specificity, ANCA detection is critical for AAV diagnosis, and ANCA positivity with a compatible clinical diagnosis usually allows the initiation of immunosuppressive treatments [8, 9].

The prompt initiation of immunosuppressive drugs to induce remission is critical for AAV patient prognosis. In generalized and severe forms, conventional induction treatment combines high doses of glucocorticoids and cyclophosphamide [10]. In addition, plasma exchange (PE) may be used in severe forms with DAH and/or severe renal involvement [11, 12]. Based on recent clinical trials, rituximab, the anti-CD20 monoclonal antibody, can be used as an alternative to cyclophosphamide. Under these regimens, AAV remission is achieved in 60–80% of the patients [13–17]. However, despite being adequately treated, some patients experience resistance to therapy or disease relapse. Moreover, a high mortality rate is observed in AAV patients, with rates reaching 10–15% within the first year following treatment initiation, the main causes of early death being infection events and vasculitis manifestations [18, 19]. Mortality rates of up to 20% after 5 years have been observed, and mortality has been shown to be higher with MPA than with EGPA and GPA [18].

To date, patients with the most severe forms of AAV—those requiring intensive care—have not been extensively and adequately analyzed. Indeed, most previous studies were small size studies, uncontrolled and monocentric. Moreover, they mingled AAV patients with manifestations related to vasculitis activity, and those with manifestations not related to it. Finally, data for AAV patients admitted to the intensive care unit (ICU) for AAV manifestations [20–24] are scarce and the prognosis of this specific population remains poorly determined.

We conducted this retrospective multicentric study to analyze disease presentation and outcome in AAV patients admitted to the ICU with acute vasculitis manifestations. Specifically, we intended to explore whether ICU admission was associated with adverse long-term outcomes. To this end, ICU-AAV patients were compared with a group of AAV patients admitted to two nephrology departments with an active disease but with no requirement of ICU care (non-ICU-AAV patients).

## Methods

### Population and inclusion criteria

We conducted a multicentric retrospective study in seventeen ICUs from French University and General Hospitals, and from Lausanne’s University Hospital in Switzerland. Inclusion criteria included: over 18 years of age, ICU admission between January 2002 and December 2012, and newly diagnosed or relapsing AAV. Only patients with acute vasculitis manifestations were included in the study. To be included, AAV initial or relapse diagnosis had to be done during the ICU stay or within the thirty days immediately prior to ICU admission.

A number of non-ICU-AAV patients were used as a control group. This group included all consecutive AAV patients of two nephrology centers (Angers and Tours University Hospitals), who were diagnosed between January 2002 and December 2012. To be included in the control group, patients had to show active newly diagnosed or relapsing AAV. Patients who required further ICU admission within the month following admission to the nephrology department were excluded from the control group.

ANCA positivity by indirect immunofluorescence (cytoplasmic or perinuclear pattern) and ELISA (proteinase-3 (PR-3) or myeloperoxidase (MPO)) was required for inclusion in both groups (ICU and non-ICU groups).

The Institutional Ethics Committees of the Angers University Hospital and Lausanne Hospital approved the study protocol (N°2013/21 and N°164/14, respectively).

### AAV diagnosis

Patients’ medical files were analyzed, and the AAV subtype (GPA, MPA and EGPA) was determined according to the European Medicines Agency vasculitis classification algorithm [25]. For newly diagnosed patients, the date of AAV diagnosis was defined as the date of ANCA determination and the date of relapse for relapsing patients was defined as the date of hospital admission. The diagnosis of new onset AAV relied on ANCA positivity and presence of vasculitis manifestations. The diagnosis of relapsing AAV was retained when it was suspected by the physician, and when the retrospective analysis of the patient’s medical history was consistent (i.e., typical clinical manifestation, increase in ANCA titer or biopsy-proven vasculitis activity).

For conflicting cases, the hospitalization report and the medical records were analyzed by 2 expert investigators (JFA and NL). If AAV activity remained doubtful, the patient was excluded from the study.

### Data collection

Patients were identified from the ICU database of each hospital. All the data were collected retrospectively by a systematic screening of patients’ medical records. The following data were collected: age, gender, height and weight, and significant aspects of past medical history. Organs affected by vasculitis were listed upon the presentation of newly diagnosed and relapsing patients. The ANCA type was recorded, and pathology data of biopsied organs were analyzed (when available) to confirm the diagnosis of vasculitis. Birmingham Vasculitis Activity Score (BVAS) 2003 was used to determine AAV activity [26].

### ICU ANCA-associated vasculitis

For ICU-AAV patients, causes of ICU admission with simplified acute physiology score II (SAPS II) and sequential organ failure assessment (SOFA) score upon admission [27, 28] were recorded. The ratio of partial pressure of arterial oxygen over inspired-fraction of oxygen (PaO_2_/FiO_2_) with ventilation initiation, the serum creatinine level and the Acute Kidney Injury Network (AKIN) score [29] upon ICU admission were used to characterize the severity of respiratory and renal injuries. Supporting therapies used during the ICU stay and their duration (mechanical ventilation, renal replacement therapy, vasopressors), septic events (as documented in the ICU hospitalization report) and death were registered. Cause of death was classified by two authors (NL and JD) after patient’s files review.

### AAV treatment

In both groups, all specific AAV induction regimens and their timing were recorded, including steroid treatment, cyclophosphamide, rituximab and plasmapheresis treatments. The use of steroid boluses, and the dosages and the number of cyclophosphamide and rituximab boluses were analyzed, as well as the number of plasma exchanges.

### Outcomes definition

For both groups, survival was analyzed until death, loss of follow-up or end of follow-up (December 2012). Survival free of end-stage renal disease of the ICU and non-ICU groups was also analyzed. Renal death was defined as the need for long-term (>3 months) renal replacement therapy.

### Statistical analysis

Quantitative parameters were presented as median (interquartile range (IQR)) and qualitative parameters as absolute number and/or percentage. Categorical and continuous data were analyzed with Chi-square (or Fisher’s exact test) and Mann–Whitney U tests, respectively. Results were presented as odd ratio (OR) with 95% confidence intervals (95 CIs); the Kaplan–Meyer method was used to analyze the survival rates of ICU and non-ICU groups. A log-rank test was used to compare the survival curves. All statistical tests were performed with a two-sided 0.05 level of significance applied. Statistical analysis was performed using SPSS software^®^ 23.0 for Macintosh and Graphpad Prism^®^.

## Results

### Baseline characteristics of the ICU and non-ICU-AAV populations

Ninety-seven and ninety-five AAV patients with a median follow-up of 2.28 [IQR 0.2–4.7] and 4.18 [IQR 1.7–7.0] years were included in the ICU group and the non-ICU control group, respectively. Characteristics of the ICU and the non-ICU groups and the main AAV manifestations upon admission are detailed in Table 1. Groups were similar regarding gender and AAV subtype, but patients of the ICU group were significantly younger. Newly diagnosed AAV was significantly more common in the non-ICU group (93%) compared with the ICU group (79%). In the ICU group, 39 patients were diagnosed during the ICU stay and 58 were diagnosed before their admission to ICU. Disease activity assessed by BVAS showed no statistical difference between groups. Heart, lung and central neurological injuries were more frequent in ICU patients than in non-ICU patients. A notably high rate of alveolar hemorrhage was observed in ICU patients (64 vs. 10% in non-ICU patients), but the renal involvement rate was comparable. ANCA subtypes by immunofluorescence and ELISA were significantly different between groups. Indeed, 62% of ICU patients had PR3-ANCAs, and 68% of non-ICU patients had MPO-ANCAs.

**Table 1:** Baseline characteristics of the ICU and non-ICU ANCA-associated vasculitis groups

	ICU-AAV (*n* = 97)	Non-ICU-AAV (*n* = 95)	*p*
Baseline characteristics			
Sex (M/F)	45/52	54/41	0.147
Age (years)	62.0 (48.0–71.0)	68.0 (55.8–75.1)	0.016
Weight (kg)	71.5 (58.0–80.0)	70.0 (60.0–82.0)	0.877
Hypertension, *n* (%)	35 (36.1)	47 (49.5)	0.061
Diabetes mellitus, *n* (%)	11 (11.3)	9 (9.5)	0.672
AAV characteristics			
Diagnosis, *n* (%)			
GPA	58 (59.8)	45 (47.4)	0.084
MPA	37 (38.1)	48 (50.5)	0.084
EGPA	2 (2.1)	2 (2.1)	0.983
ANCA type			
By immunofluorescence			
cANCA, *n* (%)	61 (62.9)	33 (34.7)	<0.001
pANCA, *n* (%)	36 (37.1)	62 (65.3)	<0.001
By ELISA			
PR3-ANCA, *n* (%)	60 (61.9)	30 (31.6)	<0.001
MPO-ANCA, *n* (%)	37 (38.1)	65 (68.4)	<0.001
Disease status, *n* (%)			
Newly diagnosed AAV, *n* (%)	77 (79.4)	88 (92.6)	0.008
Relapsing AAV, *n* (%)	20 (20.6)	7 (7.4)	0.008
BVAS	23.0 (18.0–27.5)	19.5 (15.0–31.0)	0.273
Organ involvement, *n* (%) when specified			
Cutaneous signs	25 (25.8)	19 (20.0)	0.341
Ear, nose, throat	36 (37.1)	34 (35.8)	0.849
Heart	17 (17.5)	3 (3.2)	0.001
Digestive	9 (9.3)	3 (3.2)	0.134
Lung	85 (87.6)	30 (31.6)	<0.001
Alveolar hemorrhage	62 (63.9)	9 (9.5)	<0.001
Others	23 (23.7)	21 (22.1)	0.791
Renal	83 (85.6)	87 (91.6)	0.191
Serum creatinine	256.5 (115.3–527.8)	244.0 (132.0–377.5)	0.666
Renal replacement therapy	55 (56.7)	19 (20.0)	<0.001
Neurological	25 (25.8)	12 (12.6)	0.020
Central	8 (8.2)	0 (0)	0.007
Peripheral	17 (17.5)	12 (12.6)	0.343

### Parameters specific to AAV-ICU group and assessment of organ support

The median ICU length of stay was 7 [IQR 4.5–17.5] days. Acute respiratory failure (alone or in combination with renal failure) was the main cause for ICU admission and accounted for 80% of ICU admissions. Approximately 70% of patients required respiratory assistance, which was initiated within 48 h following ICU admission in the large majority of the patients. Acute kidney injury was highly prevalent, with AKIN score ≥1 in more than 90% of patients at the time of admission, and more than half of the patients required renal replacement therapy (RRT) during their ICU stay. RRT was initiated within 48 h after admission in 35% of patients. Vasopressors were required for 25% of the patients. Infection events were reported in 40% of the patients during the ICU stay, with an identified pathogen in 82% of them. These data are summarized in Table 2.

**Table 2:** Characteristics and supportive therapies used with ICU-AAV patients

Length of stay (days)	7.0 (4.5–17.5)
Reasons for ICU admission, *n* (%)	
Respiratory failure	44 (45.4)
And renal failure	23 (23.7)
And neurological failure	1 (1.0)
Acute renal failure	17 (17.5)
And neurological failure	4 (4.1)
Neurological failure	4 (4.1)
Heart failure	3 (3.1)
Hemorrhagic shock	1 (1.0)
SOFA (at admission)	6 (4.0–9.0)
SAPS II	39.0 (31.0–51.0)
Respiratory assistance, *n* (%)	
Mechanical ventilation	
Invasive or/and noninvasive	66 (68.0)
Within 48 h of admission	58 (59.8)
Noninvasive ventilation only	19 (19.6)
Invasive ventilation only	36 (37.1)
Noninvasive and invasive ventilation	11 (11.3)
Length of respiratory assistance (days)	10.0 (5.5–18.5)
PaO_2_/FiO_2_	92.0 (58.8–182.0)
Kidney involvement	
Serum creatinine at admission, (μmol/L)	256.5 (115.3–527.8)
Maximum serum creatinine in ICU, (μmol/L)	348.0 (160.0–673.0)
AKIN score ≥1, *n* (%)	89 (91.8)
Renal replacement therapy, *n* (%)	55 (56.7)
Within 48 h of admission	34 (35.1)
Hemodynamic assistance	
Vasopressive amines, *n* (%)	26 (26.8)
Within 48 h	25 (25.8)
Length of treatment (days)	6.0 (3–11.5)
Infectious events	
Early/late*	29/10
Lung infection, *n* (%)	29 (74.4)**
Other sites, *n* (%)	10 (25.6)**
Patients with pathogen identified, *n* (%)	32 (82.1)**

* Diagnosed < or >48 h after ICU admission

**Among patients that experienced infection

Site and nature of infectious events are detailed in Additional file 1: Table 1.

### Immunosuppressive regimens

Ninety-nine percent of patients of the ICU and 98% of non-ICU groups received glucocorticoids for remission induction. Steroid pulses were given to 95 of the 97 (98%) patients in the ICU group, and to 80 of the 95 (84%) patients in the non-ICU group (*p* < 0.001). Glucocorticoids were combined with cyclophosphamide in 83 (85.6%) ICU patients and in 78 (82.1%) non-ICU patients (*p* = 0.514). PE was more frequently used in the ICU group than in the non-ICU group (*n* = 48, 54% vs. *n* = 23, 24%, respectively, *p* < 0.001).

The most common induction immunosuppressive regimen administered to the ICU group was a combination of glucocorticoids and cyclophosphamide used in 41 patients (42.3%) and a combination of glucocorticoids, cyclophosphamide and PE used in 42 patients (43.3%). In the non-ICU group, glucocorticoids and cyclophosphamide were the most frequent induction regimen (58.9% of patients). Data detailing immunosuppressive regimen used in ICU and non-ICU-AAV patients, and their timing according to ICU admission, are outlined in Additional file 2: Table 2.

### Mortality and predictors of ICU mortality

Fifteen patients (15.5%) died during the ICU stay. SAPS II and ICU SOFA scores were significantly higher in non-survivors compared to survivors. The need for mechanical ventilation (invasive or not) and vasopressors was more frequent in the non-survivor group. The requirement of RRT tended to be higher in the non-survivor group, but remained statistically not significant. Moreover, infectious events during ICU stays were significantly more prevalent in non-survivors. Non-surviving patients received cyclophosphamide more frequently than surviving patients. We did not observe any difference between survivors and non-survivors according to the timing of immunosuppressive treatment, including cyclophosphamide, with respect to ICU admission (data not shown). These data are summarized in Table 3. In a multivariate logistic analysis, cyclophosphamide was no longer associated with mortality after adjustment on SAPS II or occurrence of infection events (Additional file 3: Table 3).

**Table 3:** Comparison between survivor and non-survivor ICU-AAV patients and univariate logistic regression analysis for ICU mortality

	Survivors (*n* = 82)	Non-survivors (*n* = 15)	*p* value	Univariate logistic regression		
				OR	95% CI	*p*
Baseline characteristics						
Gender (female), *n* (%)	41 (50)	4 (26.7)	0.158	0.36	0.11–1.24	0.105
Age (years), median [IQR]	61.0 [47.0–70.3]	67.0 [65.0–75.0]	0.212	0.98	0.94–1.02	0.311
Hypertension, *n* (%)	32 (39.0)	3 (20.0)	0.158	0.39	0.10–1.49	0.169
ANCA						
c-ANCA type, *n* (%)	54 (65.9)	7 (46.7)	0.157	0.45	0.15–1.38	0.164
AAV relapse, *n* (%)	19 (23.2)	1 (6.7)	0.185	0.24	0.03–1.92	0.177
AAV involvement						
Heart, *n* (%)	14 (17.1)	3 (20.0)	0.723	1.21	0.30–4.87	0.784
CNS, *n* (%)	5 (6.1)	3 (20.0)	0.104	3.85	0.81–18.2	0.089
Digestive, *n* (%)	7 (8.5)	2 (13.3)	0.626	1.64	0.31–8.83	0.559
ENT, *n* (%)	33 (40.2)	3 (21.4)	0.159	0.37	0.10–1.41	0.147
Kidney						
Serum creatinine (µmol/L), median [IQR]	364.0 [117.5–503.0]	229.0 [104.0–682.0]	0.824	1.00	0.99–1.00	0.878
AKIN > 1, *n* (%)	74 (90.2)	15 (100)	0.351	1.51	0.17–13.1	0.706
RRT, *n* (%)	43 (52.4)	12 (80.0)	0.086	3.53	0.93–13.5	0.064
Lung, *n* (%)	71 (86.6)	14 (93.3)	0.685	2.17	0.26–18.2	0.475
DAH, *n* (%)	51 (62.2)	11 (73.3)	0.562	1.67	0.49–5.71	0.412
Respiratory assistance, *n* (%)	52 (63.4)	14 (93.3)	0.032	8.51	1.07–67.9	0.043
Infectious event, *n* (%)	26 (31.7)	13 (86.7)	<0.001	14.0	2.94–66.6	0.001
Vasopressors, *n* (%)	17 (20.7)	9 (60.0)	0.002	5.73	1.79–18.4	0.003
Plasma exchange*, *n* (%)	39 (47.6)	7 (46.7)	0.949	0.96	0.32–2.91	0.949
Cyclophosphamide*, *n* (%)	52 (63.4)	14 (93.3)	0.032	8.51	1.07–67.9	0.043
SAPS II, median [IQR]	37.5 [28.8–49.3]	52.0 [33.0–66.0]	0.006	1.06	1.02–1.10	0.003
SOFA, median [IQR]	6.0 [4.0–9.0]	8.0 [6.0–13.0]	0.040	1.21	1.04–1.41	0.013
BVAS, median [IQR]	23.0 [18.0–28.0]	21.0 [20.0–27.0]	0.916	1.01	0.94–1.08	0.899

*OR* odd ratio, *CI* confidence interval, *ANCA* anti-neutrophil cytoplasmic antibodies, *c-ANCA* cytoplasmic ANCA, *AAV* ANCA-associated vasculitis, *CNS* central nervous system, *ENT* ear nose throat, *AKIN* acute kidney injury network score, *RRT* renal replacement therapy, *DAH* diffuse alveolar hemorrhage

* Started before or during the ICU stay

The cause of ICU death was attributed to refractory vasculitis manifestations in 6 (40%) patients (DAH in 5 patients, digestive involvement in 1), to multiple organ failure likely due to sepsis in 5 (33%) patients and to neurologic causes in 4 (27%) patients, including 3 cerebral hemorrhage while receiving anticoagulation for extracorporeal membrane oxygenation.

### Long-term outcomes of ICU-AAV patients

**Fig. 1:**
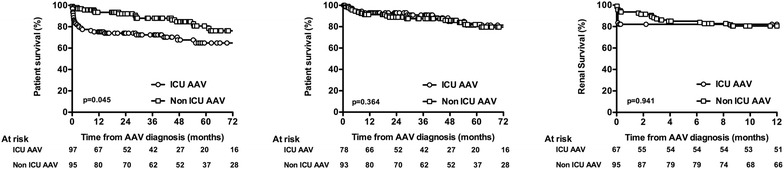
**a** Survival of ICU and non-ICU-AAV patients and **b** survival of patients who survived to the first hospital stay. **c** Renal survival of ICU and non-ICU patients. Survivals were compared using the log-rank test

Whether ICU stay can impact the prognosis of AAV patients had not been yet analyzed. Regarding hospital mortality, we observed that ICU-AAV patients had a poorer survival rate compared to non-ICU-AAV patients (Fig. 1a). Given that most deaths in the ICU group occurred soon after ICU admission, we next analyzed the long-term mortality of patients that survived to the first hospital stay (ICU or non-ICU patients). By this way, we were able to observe that the long-term mortality of AAV patients who survived to the first hospital stay was no longer different between the ICU and the non-ICU group (Fig. 1b). We also analyzed the renal outcome of groups. Long-term renal outcome was available for 67 ICU-AAV patients out of 82 and for all non-ICU-AAV patients. Survival analysis showed that renal survival was not significantly different between ICU and non-ICU patients after 1 year of follow-up (Fig. 1c).

## Discussion

In the present work, we described 97 patients who required ICU admission at AAV diagnosis or relapse. Lung involvement (notably, DAH) was the prominent cause for ICU admission, 70% of the patients requiring mechanical ventilation. Fifty percent needed RRT and 25% needed vasopressors. Comparison with a control group of AAV patients admitted to the nephrology department showed no difference in overall score of disease activity (BVAS), but ICU patients were younger and more likely to have PR3-ANCAs and GPA. A vast majority of ICU patients and non-ICU patients received an induction regimen with corticosteroid and cyclophosphamide, and ICU patients received PE more frequently. One-year mortality rate was higher in ICU patients due to high in-ICU fatality (15.5%) but, interestingly, in ICU survivors, 1-year survival after initial hospital admission was no different from non-ICU patients.

Several studies have already reported AAV patients admitted to ICU. Most included AAV patients admitted to ICU for reasons related to vasculitis manifestations and reasons unrelated to the same—such as our results cannot be readily compared. However, some of our results are in line with these reports, showing a majority of GPA among ICU-AAVs [20, 21, 30], and a high prevalence of DAH [20, 21, 24]. Previous studies have reported very variable rates of respiratory assistance (31–57%) [20, 23, 24, 31] and of RRT use (20–80%) [20, 23, 24], and variable mortality rates from 0 to 33% probably related to the heterogeneity of included patients [20, 23].

However, this study is the first to report on any long-term outcome of AAV patients with active disease after an ICU stay or to assess the impact of ICU stay in comparison with AAV patients initially admitted to non-ICU wards. Indeed, besides observing that ICU admission for acute organ dysfunction requiring organ support is associated with poor outcome, we thought that the main question was to determine the association between initial disease severity, invasive therapeutic procedures, and long-term outcome. Given that kidney involvement is a major prognostic factor in AAV [11], we estimated that AAV patients with kidney involvement constituted a pertinent control group. Unsurprisingly, a high death rate was observed with ICU patients at the early phase of the disease, but the observation that long-term outcome (both mortality and renal survival) in ICU survivors is no different from non-ICU patients is a key observation and deserves discussion. First, it can be noted that ICU patients were younger and more frequently had GPA compared with MPA, two factors related to better outcome in previous studies outside the ICU [32, 33]. The fact that GPA onset is usually earlier than MPA onset may explain the lower age and the greater frequency of patients with relapsing in the ICU group [33]. Due to the low death rate after the initial stay, no multivariate analysis to determine the association between ICU admission or not, AAV type and age with outcome was attempted. Beyond these limitations, our observation tends to show that initial disease severity, once adequately controlled by the induction regimen, is not associated with adverse long-term outcome and does not indicate a more severe disease. From an ICU point of view, it should be noted that this observation is at stake with several reports showing prolonged excess risk of death in ICU survivors in comparison with non-ICU patients [34]. Although the number of patients in our study limits the interpretation of this result, it favors the hypothesis that prolonged mortality in ICU patients is related to baseline conditions (here: the AAV disease) rather than to acute episodes [35].

Notwithstanding the relatively good prognosis with ICU survivors, ICU mortality is a concern that should be addressed. Intensity of acute organ dysfunction assessed through acute severity scores (SOFA, SAPS II and hence organ support requirement), cyclophosphamide and infection were associated with ICU death. In contrast, BVAS did not appear suitable for predicting short-term mortality of ICU-AAV patients [20]. Despite limitation of statistical analysis, cyclophosphamide did not appear as a risk factor for mortality after adjustment on SAPS II and infection occurrence. Although association between organ dysfunction and mortality is trivial in the ICU setting, the association between cyclophosphamide, infection and mortality is certainly not straightforward. Indeed, most infections were nosocomial infections in patients receiving mechanical ventilation, and the attributable mortality of such infections has been debated [36, 37]. Rather, it may represent an indicator of overall severity associated with an immunosuppressive state related to the condition which led to ICU admission (DAH and AAV), consequences of ICU care (tracheal intubation), and immunosuppressive treatments. Analysis of cause of death brings up additional relevant information, as infection causes and disease activity both represent the two major conditions associated with death. Finally, from our dataset, no straightforward message can be established for the purpose of determining whether ICU patients with AAV require a higher or lower level of immunosuppression, if this concept has any meaning.

Improved ICU outcome may come from more refined and individualized induction regimen. It has been proven that PE has benefited AAV patients with severe renal involvement (i.e., creatinine >500 μmol/L) when used in replacement of methylprednisolone boluses in AAV induction treatment, allowing the achievement of higher rates of renal recovery [11]. Moreover, evidence from clinical practice and retrospective studies also supports PE effectiveness in patients with DAH-related AAV [5, 12, 38]. However, our study does not show a clear-cut impact of PE on survival, this observation being clearly limited by the design of this study and the dataset. It should be noted that rituximab, which appeared as a novel option in both induction and maintenance regimens [14, 15] of AAV [39], was rarely used in our study. This may be explained by the date range of the study which ended in 2012, whereby rituximab was not able to pass into clinical practice. Given the limited data related to the use of rituximab in patients with severe forms of AAV including patient with DHA, The French Vasculitis Study Group recommended to use cyclophosphamide as a first-line treatment to induce remission [40]. However, recently, Cartin-Ceba et al. [41] suggested in a retrospective analysis that rituximab may be superior to cyclophosphamide to achieve remission at 6 months in AAV patients with DHA. We observed a high rate of infectious events, a majority of deaths related to DAH or sepsis in our study and an increased rate of cyclophosphamide use in non-survivors. Whether using rituximab in replacement of cyclophosphamide in this specific population may improve prognosis merits to be considered.

Our study undeniably has several limitations, starting with its retrospective design and restriction to AAV patients with ANCA positivity. Despite all efforts to be as exhaustive as possible, some data may have been missed. Given the very low prevalence of ICU admission for active AAV, a prospective study does not seem easily conceivable. We believe that the multicentric and controlled design of our study has contributed to limitation of center-dependent bias and to significant expansion of ICU-AAV-related knowledge, especially with regard to long-term prognosis of these patients.

## Conclusion

Acute respiratory failure due to DAH is the most common vasculitis manifestation which puts AAV patients in the ICU. Despite a high early ICU mortality rate, patients who survive to ICU show comparable long-term mortality and renal prognosis compared to non-ICU-AAV patients.

## Additional files

**Additional file 1: Table** **1.** Infectious events in ICU-AAV group.

**Additional file 2: Table** **2.** Induction immunosuppressive regimens of the ICU and non-ICU-AAV patients.

**Additional file 3: Table** **3.** Multivariate logistic analysis for ICU mortality.

## Abbreviations

AAV: anti-neutrophil cytoplasmic antibodies-associated vasculitis
ANCA: anti-neutrophil cytoplasmic antibodies
AKIN: acute kidney injury network score
BVAS: Birmingham vasculitis activity score
DAH: diffuse alveolar hemorrhage
EGPA: eosinophilic granulomatosis with polyangiitis
ELISA: enzyme-linked immunosorbent assay
GPA: granulomatosis with polyangiitis
ICU: intensive care unit
MPA: microscopic polyangiitis
MPO: myeloperoxidase
PaO_2_/FiO_2_: ratio of partial pressure of arterial oxygen over inspired-fraction of oxygen
PE: plasma exchange
PR-3: proteinase-3
RRT: renal replacement therapy
SAPS II: simplified acute physiology score II
SOFA: sequential organ failure assessment
