# Supplementary material for: Patients with ANCA-associated vasculitis admitted to the intensive care unit with acute vasculitis manifestations: a retrospective and comparative multicentric study
Source: Ann Intensive Care. 2017 Apr 5;7:39. doi: 10.1186/s13613-017-0262-9 (PMC5382116; doi:10.1186/s13613-017-0262-9)
Supplement: Supplementary file 1 — Additional file 1: Table 1. Infectious events in ICU-AAV group. [file 13613_2017_262_MOESM1_ESM.docx]

**Supplemental table 1.**

|  | **All (39)** | **Early (29)** | **Late (10)** | ***P**** |
| --- | --- | --- | --- | --- |
| Mean occurrence | 1.89±3.51 [0-18] | 0.48±0.78 [0-2] | 7.0±4.78 [3-18] | <0.001 |
| ICU death | 13 (33,3) | 9 (31.0) | 4 (40) | 0.704 |
| Infection site |  |  |  |  |
| Lung | 29 (74.3) | 22 (75.9) | 7 (70.0) | 0.696 |
| Septicemia | 1 (2.6) | 1 (3.4) | 0 (0) | / |
| Urinary | 3 (7.7) | 2 (6.9) | 1 (10.0) | / |
| Abdominal | 5 (12.8) | 3 (10.3) | 2 (20.0) | / |
| Undetermined | 1 (2.6) | 1 (3.4) | 0 (0) | / |
| Type of bacteria |  |  |  |  |
| Gram+ | 10 | 6 | 4 | 0.244 |
| Gram- | 22 | 16 | 6 | 1.000 |
| Undetermined | 7 | 7 | 0 | 0.158 |

* Comparisons between early and late infectious events.
