# Supplementary material for: Patients with ANCA-associated vasculitis admitted to the intensive care unit with acute vasculitis manifestations: a retrospective and comparative multicentric study
Source: Ann Intensive Care. 2017 Apr 5;7:39. doi: 10.1186/s13613-017-0262-9 (PMC5382116; doi:10.1186/s13613-017-0262-9)
Supplement: Supplementary file 2 — Additional file 2: Table 2. Induction immunosuppressive regimens of the ICU and non-ICU-AAV patients. [file 13613_2017_262_MOESM2_ESM.docx]

**Table 2 supplemental.**

|  | **ICU group** | **Non ICU group** | ***P*** |
| --- | --- | --- | --- |
| **Immunosuppressive regimens**, n (%) |  |  |  |
| Glucocorticoids-CYC | 41 (42.3) | 56 (58.9) | **0.020** |
| Glucocorticoids-PE-CYC | 42 (43.3) | 22 (23.2) | **0.003** |
| Glucocorticoids-RTX +/- PE | 3 (3.1) | 0 (0) | 0.246 |
| Glucocorticoids-PE | 4 (4.1) | 1 (1.1) | 0.368 |
| Glucocorticoids | 6 (6.2) | 13 (13.7) | 0.081 |
| Others* | 1 (1.0) | 3 (3.2) | 0.365 |
| **Treatment management**, n (%) |  |  |  |
| Glucocorticoids, started | 97 (100) | 94 (98.9) | 0.494 |
| Before ICU admission | 55 (56.7) | / | / |
| During ICU stay | 40 (41.2) | / | / |
| After ICU discharge | 2 (2.1) | / | / |
| Glucocorticoidspulses | 95 (97.9) | 80 (84.2) | **<0.001** |
| PE, started | 48 (49.5) | 23 (24.2) | **<0.001** |
| Before ICU admission | 4 (4.1) | / | / |
| During ICU stay | 42 (43.3) | / | / |
| After ICU discharge | 2 (2.1) | / | / |
| CYC, started | 83 (85.6) | 78 (82.1) | 0.514 |
| Before ICU admission | 24 (24.7) | / | / |
| During ICU stay | 42 (43.3) | / | / |
| After ICU discharge | 17 (17.5) | / | / |
| RTX, started | 3 (3.1) | 0 (0) | 0.246 |
| Before ICU admission | 1 (1.0) | / | / |
| During ICU stay | 2 (2.1) | / | / |
| After ICU discharge | 0 (0) | / | / |
| Intravenous Immunoglobulins | 8 (8.2) | 6 (6.3) | 0.606 |
|  |  |  |  |

ICU, intensive care unit ; CYC, cyclophosphamide ; RTX, rituximab ; PE, plasma exchange.

*Others: Glucocorticoids + mycophenolate mofetil or no treatment.
