# Supplementary material for: Patients with ANCA-associated vasculitis admitted to the intensive care unit with acute vasculitis manifestations: a retrospective and comparative multicentric study
Source: Ann Intensive Care. 2017 Apr 5;7:39. doi: 10.1186/s13613-017-0262-9 (PMC5382116; doi:10.1186/s13613-017-0262-9)
Supplement: Supplementary file 3 — Additional file 3: Table 3. Multivariate logistic analysis for ICU mortality. [file 13613_2017_262_MOESM3_ESM.docx]

Supplemental Table 3

|  | **Association with mortality** | | |
| --- | --- | --- | --- |
|  | **OR** | **95% CI** | ***P*** |
| **Model 1:** |  |  |  |
| Infectious event | 15.3 | 2.7-85.9 | **0.002** |
| SAPSII* | 1.06 | 1.02-1.11 | **0.004** |
| **Model 2:** |  |  |  |
| SAPSII* | 1.06 | 1.02-1.11 | **0.002** |
| Cyclophosphamide | 3.53 | 0.92-13.5 | 0.065 |
| **Model 3:** |  |  |  |
| Infectious event | 13.9 | 2.88-67.3 | **0.001** |
| Cyclophosphamide | 3.0 | 0.80-11.2 | 0.103 |

*For each unit increament
